# PHF6 recruits BPTF to promote HIF-dependent pathway and progression in YAP-high breast cancer

**DOI:** 10.1186/s12967-023-04031-8

**Published:** 2023-03-26

**Authors:** Sheng Gao, Wensheng Zhang, Jingjing Ma, Xiaojian Ni

**Affiliations:** 1 Department of Breast, Women’s Hospital of Nanjing Medical University, Nanjing Maternity and Child Health Care Hospital, Nanjing, 210004 China; 2 State Key Laboratory of Genetic Engineering, MOE Engineering Research Center of Gene Technology, Key Laboratory of Reproduction Regulation of NPFPC and Collaborative Innovation Center for Genetics and Development, Fudan University, Shanghai, 200438 China; 3 Department of General Surgery, Nanjing Drum Tower Hospital, The Affiliated Hospital of Nanjing University Medical School, Nanjing, 210004 China; 4 Department of General Surgery, Zhongshan Hospital, Fudan University, Shanghai, 200032 China; 5 Cancer Center, ZhongShan Hospital, Fudan University, Shanghai, 200032 China

**Keywords:** PHF6, BPTF, HIF, Epigenetic remodeling, Breast cancer

## Abstract

**Background:**

Aberrant epigenetic remodeling events contribute to progression and metastasis of breast cancer (Bca). The specific mechanims that epigenetic factors rely on to mediate tumor aggressiveness remain unclear. We aimed to elucidate the roles of epigenetic protein PHF6 in breast tumorigenesis.

**Methods:**

Published datasets and tissue samples with PHF6 staining were used to investigate the clinical relevance of PHF6 in Bca. CCK-8, clony formation assays were used to assess cell growth capacity. Cell migration and invasion abilities were measured by Transwell assay. The gene mRNA and protein levels were measured by quantitative real-time PCR and western blot. Chromatin immunoprecipitation (ChIP)-qPCR assays were used to investigate transcriptional relationships among genes. The Co-immunoprecipitation (Co-IP) assay was used to validate interactions between proteins. The CRISPR/Cas9 editing technology was used to construct double HIF knockout (HIF-DKO) cells. The subcutaneous xenograft model and orthotopic implantation tumor model were used to asess in vivo tumor growth.

**Results:**

In this study, we utilized MTT assay to screen that PHF6 is required for Bca growth. PHF6 promotes Bca proliferation and migration. By analyzing The Cancer Genome Atlas breast cancer (TCGA-Bca) cohort, we found that PHF6 was significantly higher in tumor versus normal tissues. Mechanistically, PHF6 physically interacts with HIF-1α and HIF-2α to potentiate HIF-driven transcriptional events to initiate breast tumorigenesis. HIF-DKO abolished PHF6-mediated breast tumor growth, and PHF6 deficiency in turn impaired HIF transcriptional effects. Besides, hypoxia could also rely on YAP activation, but not HIF, to sustain PHF6 expressions in Bca cells. In addition, PHF6 recuits BPTF to mediate epigenetic remodeling to augment HIF transcriptional activity. Targeting PHF6 or BPTF inhibitor (AU1) is effective in mice models. Lastly, PHF6 correlated with HIF target gene expression in human breast tumors, which is an independent prognostic regulator.

**Conclusions:**

Collectively, this study identified PHF6 as a prognostic epigenetic regulator for Bca, functioning as a HIF coactivator. The fundamental mechanisms underlying YAP/PHF6/HIF axis in breast tumors endowed novel epigenegtic targets for Bca treatment.

**Supplementary Information:**

The online version contains supplementary material available at 10.1186/s12967-023-04031-8.

## Background

Currently, breast cancer (Bca) has become the second most common malignancy worldwide after lung cancer, leading to an estimated annual death of 41,760 cases in women [1, 2]. Although there are effective strategies against representative Bca subtypes, such as those with abnormal overexpression of the HER2/Neu oncogene, a considerable proportion of Bca patients remain to be incurable [3]. Meanwhile, the average age of Bca patients tend to be younger in recent years. The incidence of breast cancer in city is higher than that in rural areas, and correlates with the overall education and income of women [4]. Bca is a heterogeneous disease that displays various biologic and clinical behaviors [5]. Thus, tumour heterogeneity has led to the distinct subtypes of Bca, which reveal different responses to chemotherapy and prognoses, implicating the significance of personalized treatment.

Most breast cancer cells express estrogen receptor (ERα), and drugs targeting the ERα pathway have become the mainstay of breast cancer treatment [6]. Since the early 1940s, endocrine therapy has been successfully demonstrated in patients with hormone-responsive Bca [7]. However, the majority Bca patients would inevitably suffer from drug resistance and different degrees of tumor recurrence during the middle or late stages [8]. The basic medical researches have indicated that changes of chromatin landscape or dysregulation of epigenetic factors determine the development and progression of Bca [9]. Epigenetic alterations are one of the essential mechanisms of breast tumorigenesis, including non-coding RNA, m6A methylation modification, DNA methylation modification, post-translational modification of proteins, and others [10]. Bca cells usually employ the process of epigenetic remodeling to supply the nutrients, metabolites and energy that are required for cell growth [11]. Therefore, targeting epigenetic targets of Bca has become one of the promising directions for the treatment [12]. In recent years, there is an increasing number of studies on the relationship between plant homeodomain (PHD) finger protein family members and tumors, including PHF1, PHF2, PHF6, PHF8, PHF10, and PHF19 [13]. For instance, histone lysine demethylase PHF2 is identified to be a novel regulator of the DNA damage response via modulating DNA damage-induced focus formation of 53BP1 and Bca1 [14]. The PHF2-depleted cells display genome instability and are mildly sensitive to the inhibition of poly ADP-ribose polymerase (PARP). Besides, PHF10 is the subunit of PBAF, a member of SWI/SNF family of chromatin remodelers that exert essential roles in transcriptional regulation. PHF10 interacts with MYC to promote the recruitment of PBAF complex to target gene promoters, thereby, driving MYC transcriptional activation of genes [15]. Among the PHD finger-containing protein (PHF) families, PHF6 is well-known to be a key factor in leukemia with several disease-driving mutations [16]. PHF6 is reproted to interact with histones to exert functions in leukemia, suggesting that PHF6 could play as a reader of histone modification [17]. PHF6 was also significantly elevated in liver cancer and positively correlated with TNM stage, differentiation, and lymph node metastasis of patients [18]. However, no relevant researches were reported to uncover the relationships between PHF6 and Bca.

As is well documented, hypoxic environment in Bca is significantly related to the invasive phenotype, distant metastasis, and treatment resistance of cancer cells [19]. Active type of hypoxia-inducible factor (HIF) includes an O_2_-dependent HIFα isoform, the HIF-1β subunit, and other essential cofactors. HIF mainly binds to the hypoxia-responsive elements (HREs) in the promoters of downstream targets, thereby inducing transcription of VEGFA, LOX, ANGPTL4, and others [20]. Under the normoxia condition, the oxygen-dependent prolyl hydroxylase domain (PHD) proteins and the von Hippel-Lindau (VHL) protein restrict the HIF activity via proteasomal degradation pathway [21]. Although there are three HIFα isoforms (HIF1α, HIF-2α and HIF-3α), HIF1α is regarded to be the predominant isoform. HIF1α could be mainly observed in tumor hypoxic core necrotic regions, and was overexpressed in some precursor lesions such as ductal carcinoma in situ (DCIS) [22]. Loss of the tumor suppressor genes (PTEN, TP53, or Bca1) were commonly found in Bca and activation of the PI3K/Akt/mTOR or MAPK pathways can increase HIFα transcription, translation, and protein stability independent of oxygen levels. Both human epidermal growth factor receptor 2 (HER2) and estrogen receptor alpha (ERα) could increase HIFα levels via the PI3K/Akt/mTOR crosstalk. As reproted, ERα was reported to bind directly to estrogen response elements in the HIF1α (HIF1A) promoter region to induce its levels, but not HIF2α (EPAS1) [23]. Although the angiogenesis and metabolic remodeling effects mediated by HIF mainly contribute to the progression of Bca and drug resistance, the underlying mechanisms that lead to HIF activation still remain to be elusive.

In this study, we identified that PHF6 is an oncogenic regulator in Bca that correlates with poor prognosis of patients. Mechanistically, we uncovered the potential associations between PHF6 and HIF activation. PHF6 protein could recruit BPTF to drive the expressions of HIF downstream targets. We further elucidated the upstream mechanisms of high PHF6 in Bca, highlighting epigenetic vulnerabilities in Bca treatment.

## Methods and materials

### Cell culture, virus generation, transfection

Human breast cancer cell lines (MCF-7, BT-549, and MDA-MB-231), breast epithelial cell line MCF-10A, and human embryonic kidney 293 T cells were purchased from America Type Culture Collection (ATCC, Manassas, USA). All cells were cultured with Dulbecco’s modified Eagle medium (DMEM) containing 10% fetal bovine serum (FBS, Gibco, USA) and 100 U/ml penicillin/streptomycin (ThermoFisher Scientific, USA). Cells were maintained in a humidified incubator with 5% CO_2_ at 37 °C. The lipofectamine 3000 (Invitrogen, USA) was utilized to conduct the transfection under the manufacturer’s instructions. The specific siRNA targeting PHF family members and negative controls were purchased from GenePharma (Shanghai, China). ShRNA for stable knockdown of YAP or the negative control construct using a lentiviral system, and YAP or PHF6 overexpression plasmid was constructed and validated by GenePharma (Shanghai, China). The specific shRNAs sequences targeting YAP were as the following:shYAP-1:5ʹ-GACAUCUUCUGGUCAGAGATT-3ʹ;shYAP-2:5ʹ-GGUGAUACUAUCAACCAAATT-3ʹ. shBPTF-1:5ʹ-CGCCACTAACAGAGAAGGATT-3ʹ;shBPTF-2:5ʹ-GCGGCAGCTAATGAAGAAATT-3ʹ. shPHF6-1:5ʹ-GCACCATAAGTGCATGCTCTT-3ʹ;shPHF6-2: 5ʹ-CCATTATAAGTGCATGTTGTT-3ʹ.

### Bca samples collection

The 50 paired breast cancer specimens and adjacent normal control tissues were collected from the department of General Surgery, Zhongshan Hospital, Fudan University, Shanghai from 2008 to 2020. We finally selected 35 pairs with complete clinical information. All patients have signed the informed consent and the study was approved by the Ethics Review Committee of Ethics Committee of ZhongShan Hospital, Fudan University, Shanghai.

### Generation of stable HIF1α/HIF-2α knockout Bca cell lines

In order to generate HIF-1α and HIF-2α knockouts, Bca cells (MCF-7, MDA-MB-231, BT-549) were transfected with CRISPR/Cas9 using PX459, a gift from Feng Zhang (Addgene plasmid #48139, Watertown, MA, USA) [21]. The sgRNA sequences were listed as following: HIF-1α (5ʹ-CACCGTTCTTTACTTCGCCGAGATC-3ʹ), HIF-2α (5ʹ-CACCGGCTGATTGCCAGTCGCATGA-3ʹ). Transfections were performed with Lipofectamine 2000 (Thermo Fisher, Waltham, MA, USA) and Puromycin resistant cells were detected after 3 days of puromycin selection (1 μg/mL) followed by a recovery period of two weeks. Surviving cells were single cell seeded and expanded for further analysis. Genomic DNA was extracted from the potential candidates and the targeted regions of the HIF-1α and HIF-2α genes were PCR amplified. DNA fragments were sequenced and analyzed for further selection of total HIF-1α and HIF-2α knockout clones. Western blot was performed to detect the KO efficacy of HIF1α/HIF-2α.

### Quantitative reverse transcription-polymerase chain reaction (RT-qPCR) assay

Total RNA from tissue specimens or cultured cells was purified using TRIzol reagent (Invitrogen, USA) and RNA concentration was detected. An equal amount of RNA (2 μg) was reverse transcribed into complementary DNA (cDNA) using Superscript Reverse Transcriptase (Applied Biosystems, USA). Quantitative real-time PCR (qRT-PCR) was performed on the ABI 7500 real-time PCR system (Applied Biosystems, USA) using the PowerUP SYBR Green Master Mix (Applied Biosystems, USA).

### Western blot analysis

The cultured cells were lysed using 1 × Cell lysis buffer (Cell signaling Technology, USA) containing protein inhibitor cocktail (Roche, Switzerland) and Protein concentration was measured using a BCA protein quantification kit (Pierce, USA). An equal amount of protein (20 μg) was separated by 10% SDS-PAGE and transferred onto a nitrocellulose membrane (Invitrogen, USA). The membranes were blocked with 5% non-fat milk at room temperature for one hour and then incubated with primary antibodies overnight at 4 °C. After washing with 1 × TBST, the membranes were further incubated with Horseradish Peroxidase (HRP)-conjugated secondary antibodies at room temperature for 1 h. GAPDH was used as an internal loading control. Primary antibodies used in the study were listed as the following: PHF6 (abcam, ab173304); HIF1α (abcam, ab179483); HIF2α (abcam, ab243861); BPTF (abcam, ab288159); SNF2L (Cell Signaling Technology, CST#12483); RBBP4 (abcam, ab79416); β-actin (abcam, ab8226).

### MTT, Cell Counting Kit-8 (CCK-8) and colony formation assays

The effect of PHF family members inhibition (siRNAs) on cell viability was measured by MTT assay (Sigma-Aldrich). Briefly, MCF-7 cells (2000 cells/well) were cultured in 96-well plates for 24 h or 48 h. After incubation with 20 μL of MTT solution (5 mg/ml in PBS) for an additional 4 h at 37 °C, the supernatant solution was removed, 150 μL of DMSO was added to dissolve the formazan crystal, and the absorbance of each well was measured with a microtiter reader (Tecan Infinite 200, Switzerland) at a wavelength of 490 nm. The absorbance values for control cells were set as 1 for normalization. For the CCK-8 assays, Bca cells were seeded into a 96-well plate at 2000 cells/well with 100 µl of 10% FBS DMEM. According to the protocol of CCK-8 solution (Dojindo, Kumamoto, Japan), 10 µl of CCK-8 solution diluted in 100 µl of complete culture medium replaced the original medium of each group at indicated points. After the cells were incubated in the dark at 37 °C for an additional 2 h, we detected viable cells by using absorbance at a 450-nm wavelength. For colony formation assays, we planted Bca cells into the six-well plate at a density of 1000 cells/well and then cultured them in complete culture medium at 37 °C for 14 d. After the cells were gently washed in PBS twice, they were fixed with 4% paraformaldehyde for 30 min. Then 0.1% (w/v) crystal violet was applied for staining the fixed cells for 30 min. ImageJ software (National Institute of Health, Bethesda, MD, USA) was used to count the numbers of colonies. Three independent assays were conducted to analyze cell proliferation abilities.

### Transwell migration and invasion assay

Transwell plates (24-well, pore size 8 μm (Corning, Cat.#3422)) were used for the transwell assay. The 1 × 10^5^ cells per well were seeded in the upper chamber in 100 μL of serum-free medium, and 600 μL of complete medium was added to the lower chamber as a chemoattractant at the same time. After incubated for 24 h at 37 °C, the cells remaining at the upper surface of the membrane were removed with cotton swabs, and the cells on the lower surface of the membrane are the migrated cells. After fixed with 4% paraformaldehyde and stained with 0.1% crystal violet solution, the cells that passed through the filter were photographed by microscope. The transwell invasion assay was carried out as described above, except that 100 μL of 1:8 DMEM-diluted Matrigel (BD, USA) was added to each well at 37 °C for 6 h before the cells were seeded onto the membrane, followed by incubating for 48 h.

### Luciferase reporter assay

A total of 100 ng of pGL3-Basic plasmid (Promega, Cat.#E1751) with inserts of the PHF6 promoter sequence (TSS:− 2000 ~  + 50) were co-transfected into MDA-MB-231 and MCF7 cells using Lipofectamine^®^ 2000 transfection reagent (Thermo Fisher, Cat. #11668019) along with 200 ng of YAP/YAP-mutant construct and 10 ng of Renilla luciferase pRL-TK plasmid (Promega, Cat.#E2241). After 48 h, the dual luciferase assay was performed using the Dual-Luciferase^®^ Reporter Assay System (Promega, Cat. #E1910), under the manufacturer’s protocol. Luciferase activity was detected as the ratio of firefly luciferase signal to Renilla luciferase signal. All measurements were normalized to the control group alone. Each experiment was performed in triplicate.

### Chromatin immunoprecipitation (ChIP)-qPCR assay

Cells were exposed to 20% or 1% O_2_ for 24 h, crosslinked with 1% formaldehyde.for 10 min at room temperature, and quenched in 0.125 M glycine. Cells were lyzed in buffer (50 mM Tris–HCl, 10 mM EDTA, 1% SDS, protease inhibitor cocktail), sonicated, and subjected to immunoprecipitation overnight in the presence of salmon sperm DNA/protein A beads with antibodies against PHF6, HIF-1, RNA BPTF, or IgG at 4 °C. Precipitated chromatin DNA was extensively washed, eluted with freshly prepared elution buffer (0.1 M NaHCO3, 1% SDS), decrosslinked at 65 °C for 4 h followed by treatment with proteinase K at 45 °C for 45 min, purified with phenol/chloroform/isoamyl alcohol (25:24:1, v/v), and quantified by real-time qPCR assay.

### Co-immunoprecipitation (IP) and immunoblot assays

For immunoprecipitation, protein was extracted by HEPES lysis buffer (20 mM HEPES, 150 mM NaCl, 1 mM EDTA, 0.5% NP-40, 0.25% deoxycholate) with protease-inhibitor cocktail (Roche). Briefly, the lysates were incubated with 20 protein A/G-conjugated agarose beads (Roche) and 5 mg antibody or anti-flag beads (Sigma) at 4 ℃ overnight. After washed 3 times by PBST, beads were heated with SDS-loading and analyzed by western blotting.

### Database analysis

The TCGA-Bca cohort were obtained from The Cancer Genome Atlas (TCGA, https://tcga-data.nci.nih.gov/tcga/). The expression levels of PHF6 and its correlation with the clinical characteristics of breast cancer patients were analyzed. The expression of PHF6 mRNAs was normalized to Log2 counts and then analyzed. Kaplan–Meier survival analysis and log-rank tests were performed using the OS and RFS data. The Meta-validation Bca cohort containing 2032 samples was derived from the Kaplan–Meier Plotter dataset (http://kmplot.com/analysis/index.php?p=service&cancer=breast).

### Xenograft tumor model

Female BABL/c nude mice (6-week old) were obtained from the Shanghai SLAC Animal Center (Shanghai, China) and randomly assigned into indicated groups. 2 × 10^6^ cells in 100 μL PBS/Matrigel (1:1, Corning) were injected into the second left mammary fat pad of female NSG or NOD/SCID mice (6–8 weeks old). Mice that were implanted with MCF-7 cells were administrated subcutaneously with a slow-release 17-estradiol pellet (0.72 mg/60-day release/pellet) one day before cell implantation. Tumor size was measured every week and tumor volume was calculated (length × width2 × 1/2). Primary tumors were harvested, photographed, and weighed. In vivo animal experiments were reviewed and approved by the Experimental Animal Ethics Committee of ZhongShan Hospital, Fudan University, Shanghai.

### Statistical analysis

All experimental results were presented as Mean ± Standard deviation. The differences between groups were analyzed using Student’s t-test or one-way ANOVA. Kruskal–Wallis (K-W) test was used to assess associations between PHF6 and clinical features. The OS and RFS were analyzed using the Kaplan–Meier curves and compared by the log-rank test. The *P* < 0.05 was considered to be statistically significant. All statistical analysis was conducted by GraphPad Prism (V8.0, Prism, USA).

## Results

### PHF6 promotes colony formation, migration and invasion of breast cancer cells in vitro

**Fig. 1:**
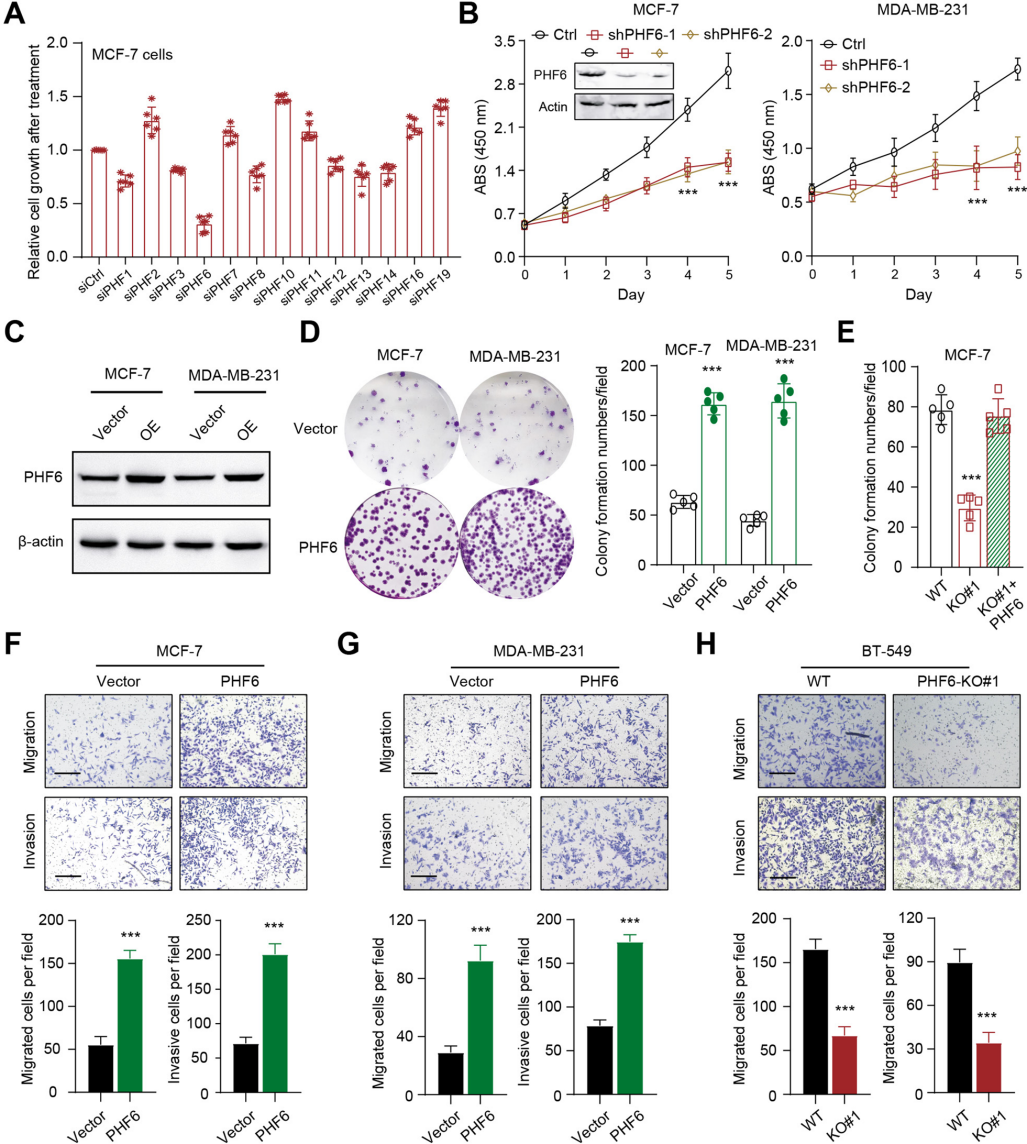
Identification of PHF6 as a required epigenetic regulator for Bca cell growth and progression. **A** The MTT assay revealing the siRNA KD of 13 candidate PHF family genes and their effects on the growth of MCF-7 cells. Quantitative data shown are representative of 5 experiments. **B** CCK-8 assays were further used to compare the cell growth abilities in control and shPHF6 groups at the indicated time points. Western blotting assay showed the PHF6 proteins in control and PHF6-KD groups. **C** Western blotting assay showed the overexpression of PHF6 in Bca cells. **D** PHF6 overexpression promotes the colony formation abilities in Bca cells. Quantification data was shown on the right in the indicated groups. **E** The colony formation ability of cells was abolished with PHF6 deficiency, and rescued by ectopic overexpression of PHF6. **F**, **G** Assessment of migration and invasion abilities in control and PHF6-overexpressing MCF-7 (F) and MDA-MB-231 (G) cells. Quantification data was shown on the below. **H** Assessment of migration and invasion abilities in parental control and PHF6-deficient BT-549 cells. Quantification data was shown on the below. *P < 0.05, **P < 0.01, ***P < 0.001

To elucidate the roles of PHF family proteins in human breast cancer progression, we conducted the MTT screen assays using siRNAs targeting specific members in MCF-7 cells. Relative to other proteins, PHF6 was the most potent hit (Fig. 1A and Additional file 1: Fig S1A). The siRNA sequences targeting each PHF family member were listed in Additional file 2: Table S1. Besides, we utilized the lentivirus infection technology to knock down PHF6 levels. PHF6 inhibition markedly restricted the in vitro cell growth relative to control cells, as evidenced by the CCK-8 assays in two cell lines (Fig. 1B). FLAG-PHF6 plasimids were transfected into cells to construct the PHF6-overexpressing cells (Fig. 1C). PHF6 overexpression significantly promoted cell colony growth of MCF-7 and MDA-MB-231 cells, individually (Fig. 1D-E). In contrast, PHF6 deficiency inhibited cell gowth, but ectopic expression of PHF6 completely rescued the impaired colony formation abilities (Fig. 1F). To determine specificity of PHF6 overexpression on cell metastatic potentiality, we utilized the PHF6-overexpressing MCF-7 and MDA-MB-231 cells to confirm that PHF6 could notably enhance cell migration and invasion abilities (Fig. 1F-G). Lastly, PHF6 loss could largely inhibit migration and invasion abilities of BT-549 cells. Collectively, we identified that the epigenetic regulator PHF6 is required for the tumorigenesis of breast cancer.

### PHF6 physically interacts with HIF-1 and HIF-2 to enhance HIF transactivation

**Fig. 2:**
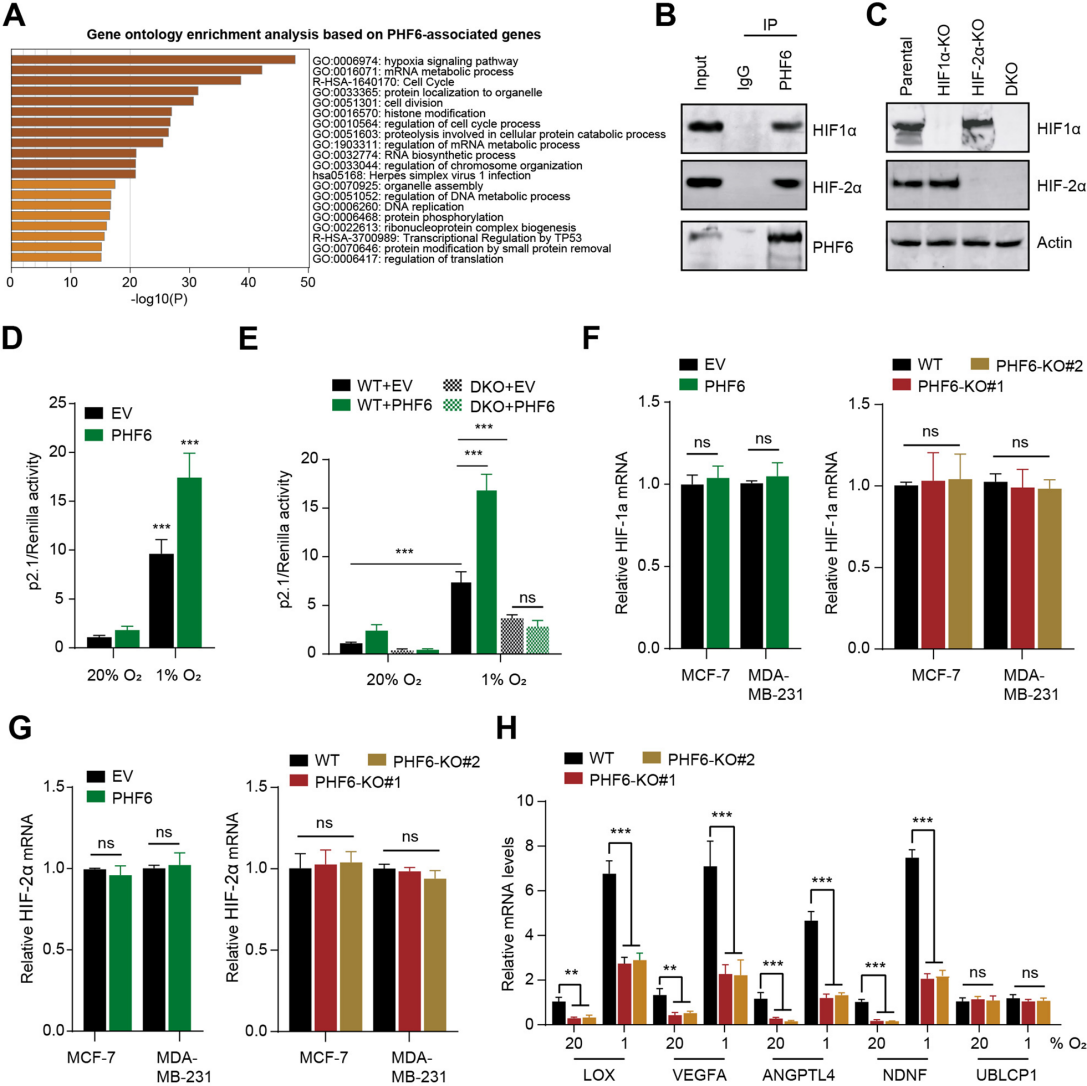
PHF6 physically interacts with HIF-1 and HIF-2 to enhance HIF transactivation. **A** The Gene Ontology (GO) bioinformatic analysis revealing the enriched items in breast cancer patients with upregulated PHF6 expression. **B** The Co-Immunoprecipitation (Co-IP) assay showing the endogeous interactions between PHF6 and HIF(HIF-1α, HIF-2α) proteins. **C** Immunoblot assays of indicated proteins in parental, HIF-1α–KO, HIF-2α–KO, or HIF-1/2α–DKO MDA-MB-231 cells exposed to 1% O_2_ for 24 h (N = 3). **D** MCF-7 cells were co-transfected with a HIF-1 luciferase reporter (p2.1), pSV-Renilla, and vector encoding WT or FLAG-PHF6 or EV. After exposed to 20% or 1% O_2_ for 24 h, cells were subjected to dual-luciferase reporter assays. **E** The p2.1, pSV-Renilla, and vector encoding FLAG-PHF6 or EV plasmids were co-transfected into WT and HIF-DKO MCF-7 cells. After exposed to 20% or 1% O_2_ for 24 h, cells were subjected to dual-luciferase reporter assays. **F** The qPCR assays detecting the associations between HIF-1α and PHF6. **G** The qPCR assays detecting the associations between HIF-2α and PHF6 in Bca cells. **H** The RT-qPCR analysis detecting the indicated mRNAs in parental or PHF6 KO MCF-7 cells that were exposed to 20% or 1% O_2_ for 24 h. *P < 0.05, **P < 0.01, ***P < 0.001

To further investigate how PHF6 regulates breast cancer progression, we performed the correlation analysis and identified the top 500 genes with the highest PHF6 correlation coefficient based on the transcriptome data of TCGA-Bca cohort. The Gene Ontology (GO) bioinformatic results indicated that hypoxia signaling was significantly enhanced in breast cancer patients with upregulated PHF6 expression (Fig. 2A). Given the tight associations between PHF6 and hypoxia signaling, we speculated whether PHF6 physically interacts with HIF-1 and HIF-2 in human breast cancer cells. After the MCF-7 cells were exposed to 1% O_2_ for 12 h, the whole cell lysates were subjected to the Co-Immunoprecipitation (Co-IP) assay with anti-PHF6 antibody or control antibody IgG. The Co-IP assay demonstrated the physical interactions between endogenous HIF1α/2α and PHF6 in hypoxic breast cancer cells (Fig. 2B). Besides, we conducted the HIF luciferase reporter assay to further elucidate whether PHF6 could enhance HIF transcriptional ability. The HIF luciferase reporter (p2.1), pSV-Renilla, and vector or FLAG-PHF6 expression vector were co-transfected into the MCF-7 cells that were further cultured under 20% or 1% O_2_ for 24 h. Cells with knockout (KO) of HIF-1α, HIF-2α, or both via the CRISPR/Cas9 technology were stably generated and the western bot was used to detect HIF-1/2α proteins under the hypoxia condition (Fig. 2C). The dual-luciferase reporter assays revealed that ectopic expression of PHF6 could notably promote HIF luciferase reporter activity in hypoxia-treated MCF-7 cells, relative to cells in vector control group (Fig. 2D). However, the PHF6’s effect on HIF transcriptional activity in hypoxia-treated cellswas completely abolished by HIF1α/HIF-2α double knockout (DKO), suggesting the specific PHF6-meidated coactivation of HIF1α/HIF-2α (Fig. 2E). To rule out the potential possibilities that PHF6 could directly influence HIF1α/HIF-2α levels, we detected that neither PHF6 overexpression nor PHF6 ablation could alter the mRNA levels of HIF1α/HIF-2α (Fig. 2F-G). Lastly, the specific HIF downstream hits, LOX, VEGFA, ANGPTL4, and NDNF, were notably decreased in PHF6-deficient MCF-7 cells treated with hypoxia, as indicated by the RT-qPCR analysis (Fig. 2H). Nevertheless, PHF6 deficiency failed to alter the mRNA levels of non-HIF target gene UBLCP1 under the hypoxia condition (Fig. 2H). Taken together, these results implicated that PHF6 could modulate a list of HIF target genes under hypoxia in breast cancer.

### PHF6 recruits BPTF to activate HIF target genes in breast cancer cells

**Fig. 3:**
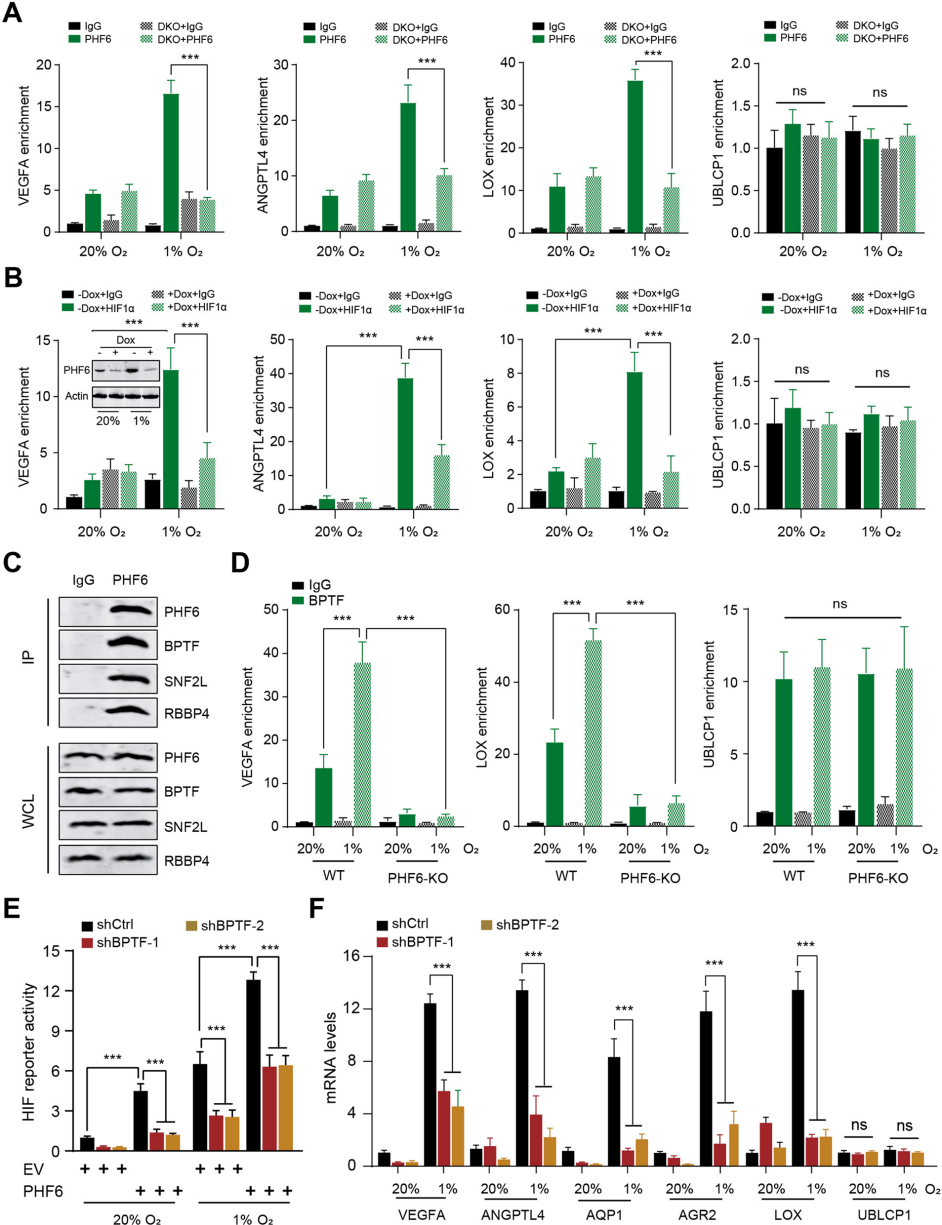
PHF6 recruits BPTF to activate HIF target genes in breast cancer cells. **A** The ChIP-qPCR assay was conducted with PHF6 antibody in parental and HIF-DKO MCF-7 cells which were exposed to 20% or 1% O2 for 24 h. **B** The ChIP-qPCR assay was conducted with HIF-1α antibody in control and doxycycline (DOX)-inducible PHF6 knockdown cells which were exposed to 20% or 1% O2 for 24 h. PHF6 proteins were shown by western blotting assay in the indicated groups. **C** The Co-Immunoprecipitation (Co-IP) assay showing the endogeous interactions between PHF6 and NURF subunits (BPTF, SNF2L, RBBP4). **D** The ChIP-qPCR assays using BPTF antiboy or IgG control in WT and PHF6-KO MCF-7 cells that were exposed to 20% or 1% O_2_ for 24 h. **E** The HIF luciferase reporter activity was detected in MCF-7 cells derived from the indicated groups that were cultured in 20% or 1% O_2_ for 24 h. **F** The RT-qPCR analysis of mRNAs of HIF targets and non-HIF target UBLCP1 in shCtrl and shBPTF MCF-7 cells that were exposed to 20% or 1% O_2_ for 24 h. *P < 0.05, **P < 0.01, ***P < 0.001

To further figure out the roles of PHF6 in HIF-1 binding to the HRE, we conducted the ChIP-qPCR assay in MCF-7 cells that were exposed to 20% or 1% O_2_ for 24 h. Apparently, PHF6 occupied the chromatin HRE regions of VEGFA, ANGPTL4, and LOX, relative to control IgG (Fig. 3A). Of note, hypoxia treatment could notably enhance PHF6 occupancy on the indicated HIF targets by 2.5–3.5 folds, apart from the non-HIF target UBLCP1 (Fig. 3A). Besides, we further utilized the HIF1α/HIF-2α DKO cells to investigate whether HIF1α/HIF-2α are required for PHF6 binding to HIF downstream hits. The ChIP-qPCR assays were conducted to compare the PHF6 occupancy in WT and HIF-DKO MCF-7 cells that were cultured in 20% or 1% O_2_ condictions for 24 h, individually. We found that HIF1α/HIF-2α loss notably impaired the PHF6 occupancy on VEGFA, ANGPTL4, LOX, but not the UBLCP1 (Fig. 3A). Collectively, these findings suggested that PHF6 coordinates HIF1α/HIF-2α to bind to HIF downstream targets in breast cancer cells. Next, we also generated doxycycline (DOX)-inducible PHF6 knockdown in MCF-7 cells and exposed them to 20% or 1% O_2_ for 24 h, respectively. As expected, the ChIP-qPCR results revealed that PHF6 KD could significantly abolish HIF-1 occupancy at HREs of indicated genes under hypoxia (Fig. 3B). Both hypoxia and PHF6 KD could not alter HIF-1 enrichment on the control gene UBLCP1 (Fig. 3B). These results indicated the mutual recruitment of PHF6 and HIF-1 to HIF target genes in breast cancer cells under hypoxia.

Our previous Co-IP assays discovered that PHF6 could directly interact with subunits of NURF complex, including BPTF, SNF2L, and RBBP4 (Fig. 3C). Previous studies have already indicated that BPTF could alter the chromatin configurations and elevate the local accessibility to drive downstream targets. Thus, we wondered whether PHF6 could recruit BPTF to sustain HIF transcriptional activity. Firstly, the ChIP-qPCR assay revealed that BPTF could bind to the HREs of HIF targets under hypoxia, but not the non-HIF target UBLCP1 (Fig. 3D). The Ctrl or BPTF-KD MCF-7 cells were transfected with HIF reporter plasmid, pSV-Renilla, and PHF6-FLAG vector or EV, and exposed to 20% or 1% O_2_ for 24 h. Consistent with the effect of PHF6 KD, BPTF-KD1 or KD2 could notably decrease HIF transcriptional activity in MCF-7 cells treated with hypoxia. Furthermore, the PHF6-mediated HIF activation was also dramatically impaired by BPTF KD in both non-hypoxic and hypoxic breast cancr cells, as indicated by HIF luciferase reporter assays in MCF-7 cells (Fig. 3E). Consistently, BPTF-KD1 or KD2 decreased the HIF targets (VEGFA, ANGPTL4, AQP1, AGR2, LOX) in both non-hypoxic and hypoxic MCF-7 cells, but not the UBLCP1 (Fig. 3F). Collectively, BPTF promotes HIF transcriptional activity and is required for PHF6-mediated HIF activation.

### Upstream YAP signals govern the high PHF6 expressions in breast cancer cells

**Fig. 4:**
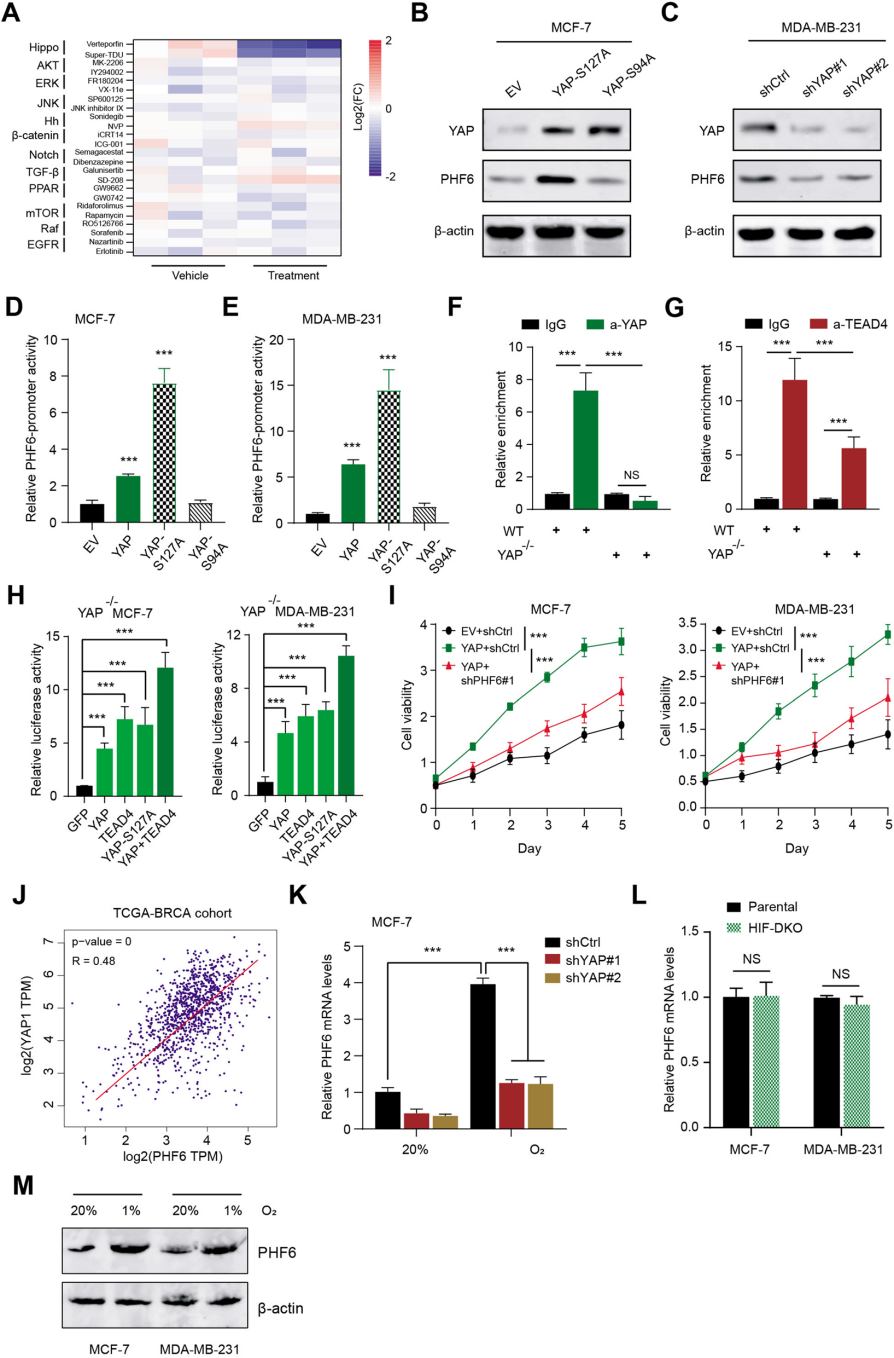
YAP signals govern the high PHF6 expressions in breast cancer cells. **A** The RT-qPCR analysis of PHF6 mRNAs in MCF-7 cells treated with DMSO and indicated compounds targeting various signals. The levels were normalized and illustrated by heatmap. **B** Western blotting assay detecting the PHF6 proteins in MCF-7 cells transfected with EV, YAP-S127A and YAP-S94A plasmids. **C** Western blotting assay detecting the PHF6 proteins in shCtrl and shBPTF MDA-MB-231 cells. **D**, **E** Dual luciferase reporter assays showed that PHF6 promoter activity in MCF-7 (D) and MDA-MB-231 (E) cells tranfected with EV, YAP, YAP-S127A and YAP-S94A plasmids. **F** ChIP-qPCR assay was conducted with a-YAP antibody and control IgG to show YAP binding to PHF6 promoter in parental and YAP-deleted cells. **G** ChIP-qPCR assay was conducted with a-TEAD4 antibody and control IgG to show TEAD4 binding to PHF6 promoter in parental and YAP-deleted cells. Besides, YAP loss impaired TEAD4 binding ability. **H** Detection of relative luciferase activity in the indicated YAP-deleted MCF-7 cells transfected with EV-GFP, YAP, TEAD4, YAP-S127A and YAP + TEAD4, respectively. **I** CCK-8 assays were utilized to determine cell growth rates in the indicated groups, including EV + shCtrl, YAP + shCtrl, and YAP + shPHF6#1. MCF-7 cells (left), MDA-MB-231 cells (right). **J** Correlation analysis showing the relationships between PHF6 and YAP1 levels in TCGA-Bca cohort. **K** The RT-qPCR analysis of PHF6 mRNA levels in shCtrl and shYAP MCF-7 cells that were exposed to 20% or 1% O_2_ for 24 h, individually. **L** The RT-qPCR analysis of PHF6 mRNA levels in parental and HIF-DKO Bca cells. **M** Western blotting assays were used to detect PHF6 proteins in Bca cells under 20% or 1% O_2_ for 24 h, individually. *P < 0.05, **P < 0.01, ***P < 0.001

To discover the upstream signals that govern PHF6 expression to possibly impact hypoxia signaling, we conducted a screening in MCF-7 cells based on a panel of compounds that block multiple signals, such as Hippo, AKT, ERK, β-catenin, and others. As compared to other signals, YAP signal inhibition by verteporfin or Super-TDU treatment could induce more 60% reductions of PHF6 mRNA levels (Fig. 4A). Subsequently, YAP-S127A and YAP-S94A mutants were transfected individually, and the results indicated that YAP-S127A remarkably elevated PHF6 expressions in MCF-7 cells, as compared to the defective YAP1-S94A mutant (Fig. 4B). Besides, YAP KD-1 and KD-2 could reduce the PHF6 expressions (Fig. 4C). The dual luciferase reporter assay further revealed that YAP overexpression could significantly increase PHF6 promoter activity in breast cancer cells, which was even higher induced by YAP-S127A (Fig. 4D, E). However the defective YAP1-S94A mutant failed to exert the effect (Fig. 4D, E). In addition, ChIP-qPCR assay also demonstrated the co-occupancy of YAP and TEAD4 on the promoter region of PHF6, whereas YAP loss impaired the TEAD4 binding to the promoter (Fig. 4F, G). Consistently, as compared to GFP control group, the constitutively activated YAP mutant (YAP-S127A) in YAP-deleted cells could notably elevate the PHF6 promoter activity, which was further enhanced with the co-transfection of YAP and TEAD4 (Fig. 4H). Meanwhile, we generated YAP-overexpressing MCF-7 and MDA-MB-231 cells and utilized the shRNA lentivirus to knockdown PHF6. The CCK-8 assays indicated that YAP could enhance cell growth, whereas PHF6 KD attenuated the YAP-activated effect (Fig. 4I). The positive correlation between PHF6 and YAP was further validated in the TCGA-Bca cohort with *Pearson’s r* = 0.48 (Fig. 4J). Previous documents revealed that hypoxia could mediate YAP activation, and we observed that hypoxia treatment (1% O_2_) could notably elevate PHF6 expressions (Fig. 4K). We further generated YAP KD-1 and KD-2 MCF-7 cells which were exposed to 20% or 1% O_2_ for 24 h, whereas hypoxia could hardly elevate PHF6 levels in YAP-KD cells (Fig. 4K). However, HIF-DKO hardly induced alterations of PHF6 mRNA levels, indicating that PHF6 is not the direct target of HIF (Fig. 4L). PHF6 proteins were notably elevated in MCF-7 or MDA-MB-231 cells under hypoxia (Fig. 4M). Thus, hypoxia could increase PHF6 proteins independent of HIF-1/2. Collectively, these data suggested that hypoxia could rely on YAP activation, but not HIF, to sustain PHF6 expressions in breast cancer cells.

### PHF6 depends on HIF crosstalk to drive progression of Bca cells in vitro and in vivo

**Fig. 5:**
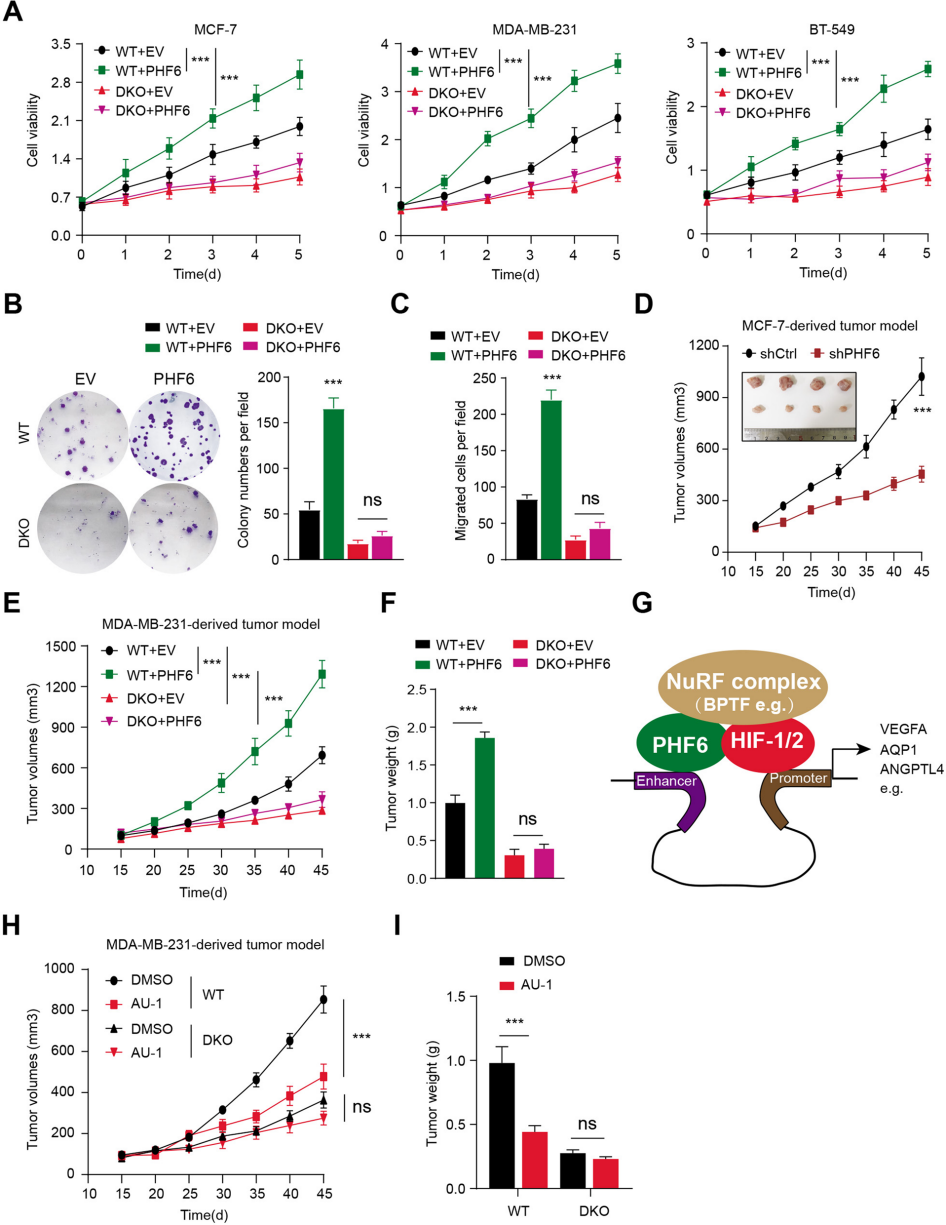
PHF6 depends on HIF crosstalk to drive progression of Bca cells in vitro and in vivo. **A** Parental and HIF-DKO Bca cells (MCF-7, MDA-MB-231, BT-549) expressing EV or PHF6 were subjected to conduct the CCK-8 assays. **B** Colony formation assays were conducted in Parental and HIF-DKO MCF-7 cells expressing EV or PHF6. Quantification data was shown on the right. **C** Cell migration assays were conducted in Parental and HIF-DKO MCF-7 cells expressing EV or PHF6. Quantification data was shown. **D** The subcutenous xenograft tumor model was conducted the compare the differential growth rates in tumors drived from shCtrl and shPHF6 cells. **E** Parental and HIF-DKO MDA-MB-231 cells expressing EV or PHF6 were orthotopically implanted into the mammary fat pad of female NOD/SCID mice, respectively. The corresponding tumor growth curves were generated. **F** The corresponding tumor weights from the indicated groups was compared. **G** Illustration of PHF6/BPTF/HIF regulatory mechanisms in driving HIF downstream targets. **H**, **I** The orthotopic implantation tumor model showing the AU1 responses of tumors derived from the parental and HIF-DKO MDA-MB-231 cells. Quantification of tumor volumes and weight were shown in (I) and (J). *P < 0.05, **P < 0.01, ***P < 0.001

To investigate whether HIF is required for PHF6-mediated breast tumor progression, we thus established parental and HIF1α/HIF-2α DKO breast cancer cells overexpressing FLAG-PHF6 or EV. The CCK-8 assays indicated that PHF6 could notably elevate cell growth rates, which was completely abolished by HIF1α/HIF-2α DKO in three independent cell lines (Fig. 5A). Besides, PHF6 overexpression could further enhance cell colony formation ability, but HIF1α/HIF-2α DKO completely impaired the effect (Fig. 5B). The similar results were also observed in MDA-MB-231 cells, where HIF1α/HIF-2α DKO completely abolished the PHF6-activated migration ability (Fig. 5C). The subcutenous xenograft tumor model indicated that PHF6 KD could restrict the in vivo tumor growth, as indicated by tumor volumes (Fig. 5D). The cells from the above four groups were further implanted into the mammary fat pad of female NOD/SCID mice. Apparently, enhanced PHF6 expressions in MDA-MB-231 cells significantly promoted in vivo tumor growth in mice, which was further abolished by HIF1α/HIF-2α DKO (Fig. 5E, F). Given that PHF6 recruits BPTF to sustain HIF transcriptional activity, we thus wondered whether targeting BPTF using specific inhibitor (AU1) could suppress breast cancer progression. We utilized the parental and HIF1α/HIF-2α DKO MDA-MB-231 cells to construct the orthotopic implantation tumor model and found AU1 could notably suppress the tumor growth in mice, as compared to tumors derived from mice in DMSO group (Fig. 5H, I). However, AU1 could hardly inhibit tumor progression in tumors derived from the HIF1α/HIF-2α DKO cells, suggesting that AU1 depends on HIF to exert the anti-tumor effect (Fig. 5H, I). Taken together, these data implicated that PHF6 depends on HIF signaling to potentiate breast cancer progression in vitro and in vivo*.* In addition, targeting PHF6/BPTF creates an epigenetic vulnerability for breast cancer treatment.

### PHF6 correlates with HIF-signature in Bca samples that has clinical significance

**Fig. 6:**
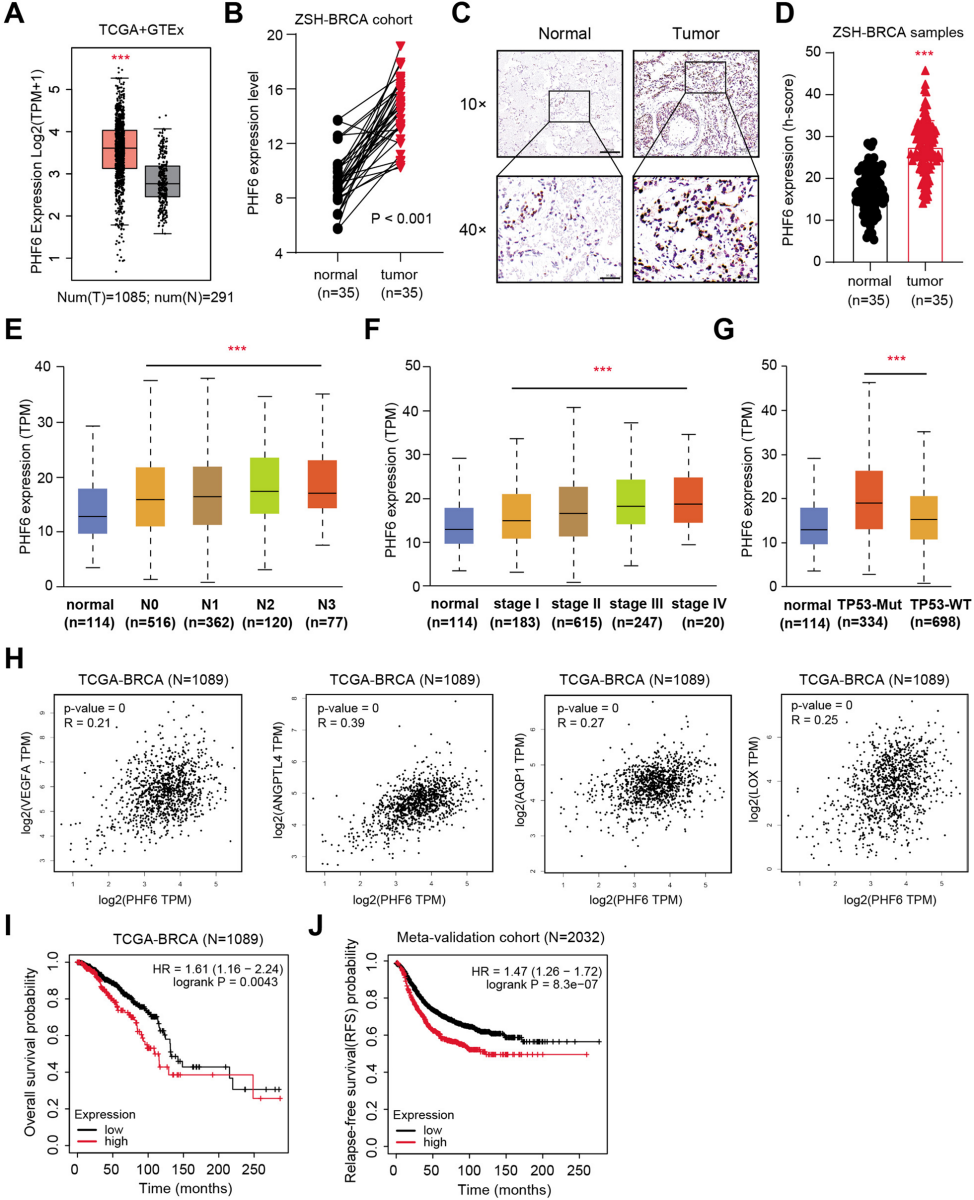
PHF6 possesses clinical significance and correlates with HIF-signature in Bca samples. **A** Differential analysis based on TCGA-Bca indicates the PHF6 levels in tumor and normal samples and was shown by boxplot. **B** The relative expression of PHF6 in breast cancer tissues or normal control tissues from the collected ZSH-Bca cohort was analyzed by qRT-PCR (n = 35, *P* < 0.001). **C** Representative PHF6 immunohistochemical (IHC) staining in breast cancer tissues and adjacent normal tissues. Scale bar = 200 μm (upper), and 50 μm (lower). **D** Quantification of PHF6 IHC-scores in tumor and normal samples. **E**, **G** Kruskal–Wallis (K-W) test showing the relationships between PHF6 and hazard factors, like lymphatic stages (E), clinicalpathological status (F) and TP-53 mutation (G). **H** Correlation analysis was conducted to uncover relationships between PHF6 and HIF downstream targets based on TCGA-Bca cohort, including VEGFA, ANGPTL4, AQP1, and LOX. **I**, **J** Kaplan-Meir analysis with log-rank test was performed to assess the prognostic value of PHF6 in large Bca samples, including TCGA-Bca cohort (N = 1089, I) and external Meta-Validation Bca cohort (N = 2032, J). *P < 0.05, **P < 0.01, ***P < 0.001

**Fig. 7:**
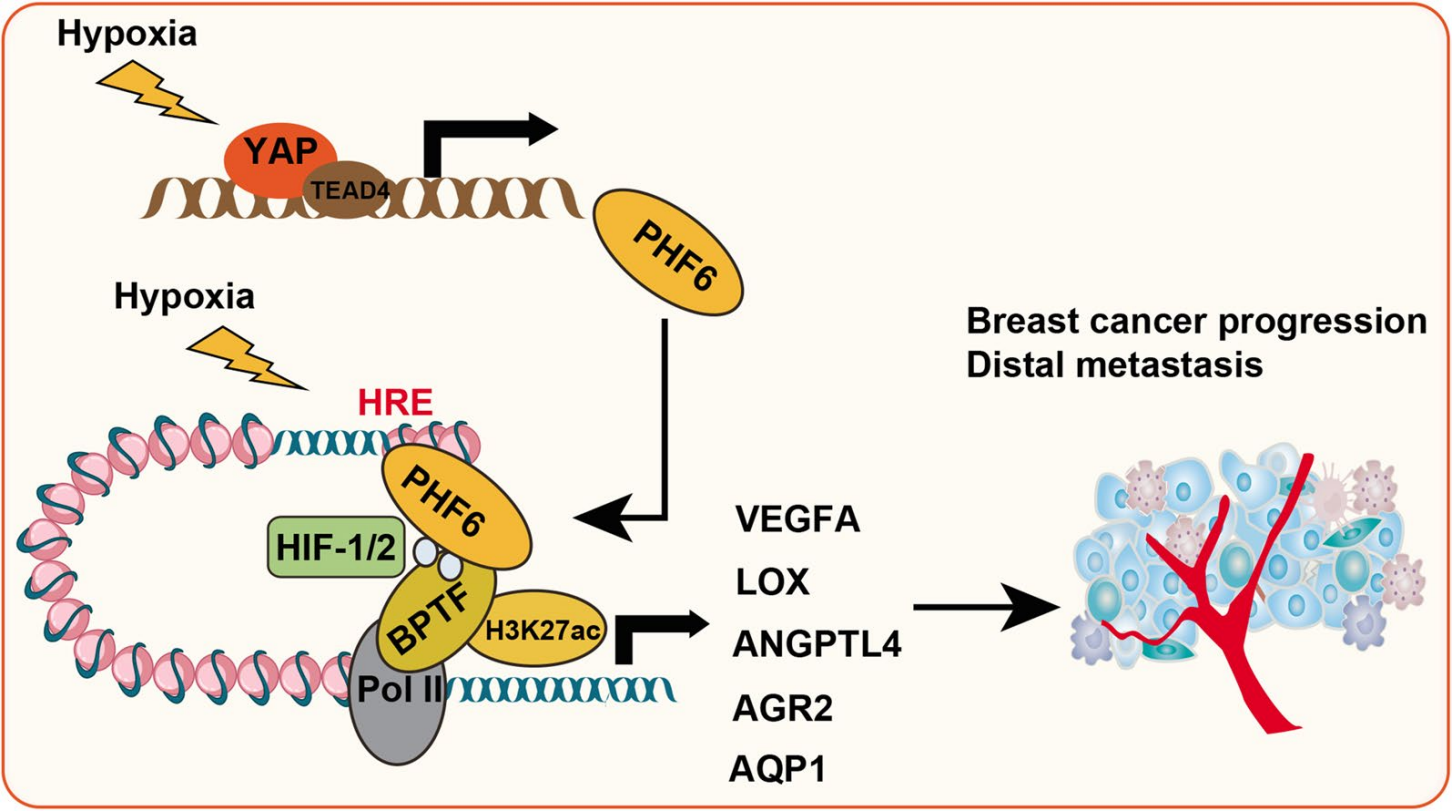
The graphical illustration of YAP/PHF6/HIF axis in amplifying HIF signaling under hypoxia that promotes tumour growth and distal metastasis of Bca

Considering that PHF6 is a required regulator that drives HIF signaling in breast cancer progression, we thus want to further evaluate its prognostic significance in breast cancer. First of all, we queried the expression data of PHF6 in TCGA-Bca dataset and found that PHF6 was notably increased in tumors versus normal tissues (Fig. 6A). Meanwhile, we observed that breast cancer tissues had notably higher mRNA levels of PHF6 in the ZSH-Bca cohort via qPCR assay (Fig. 6B,  = 35, *P* < 0.001). Accordingly, we conducted the immunohistochemistry (IHC) assay in the collected samples and confirmed the higher protein levels of PHF6 in tumor versus normal tissue sections (Fig. 6C, D). In addition, we downloaded the clinical information of patients derived from the TCGA-Bca cohort, including TNM stages, TP-53 mutation status, pathlogical stages. We performed the Kruskal–Wallis (K-W) test and found that PHF6 expressions were positively associated with clinicalpathlogical stages, lymphatic metastasis and TP53-mutation genetic phenotype (Fig. 6E, G). Considering the tight relationships between PHF6 and HIF signaling, we calculated the *Pearson* correlation coefficients in 1089 TCGA-Bca samples and found the positive associations between PHF6 and HIF downstream targets (VEGFA, ANGPTL4, AQP1, LOX) in Fig. 6H. Consistently, we also conducted the prognostic analysis and found that Bca patients with high PHF6 had shorter overall survival (OS) months than those with low PHF6 levels based on the data of 1089 patients in TCGA-Bca cohort (log-rank test *P* = 0.0043, Fig. 6I). We downloaded and merged the PHF6 data from multiple datasets based on the bioinformatics tool Kaplan–Meier Plotter (http://kmplot.com/analysis/index.php?p=service&cancer=breast). We conducted Kaplan–Meier analysis in the Meta-validation cohort and observed that patients with high PHF6 had shorter relapse-free survival (RFS) time than those with low PHF6, in line with the notion that PHF6 correlates with tumor recurrence (log-rank test *P* = 8.3e − 07, N = 2032, Fig. 6J). Last of all, we illustrated the clinical significance of the YAP/PHF6 axis in promoting Bca HIF signaling under the hypoxia condition (Fig. 7). Taken together, these data implicated that PHF6 is an epigenetic regulator that possesses prognostic significance.

## Discussion

It is clear that epigenetic alterations have represented a molecular hallmark in breast cancer tumorigenesis [24]. Owing to intensive researches performed in epigenetic field, effective approaches have continuously identified novel biomarkers to predict breast cancer prognosis and the potentiality of epigenetic therapies [25]. For instance, Detection of epigenetic regulators, such as ZMYND8, E2F1 and KDM6A, would be beneficial for determining prognosis and management in breast cancer patients [26–28]. There is an essential need to improve our perspectives in epigenetic mechanisms of breast cancer tumorigenesis and its clinical outcome. In the current study, we intended to uncover the potential relationships between PHF family members and Bca aggressiveness. Firstly, we designed a MTT screen assay to find that PHF6 is most potent hit and targeting PHF6 repressed the largest cell growth relative to other members. Subsequently, experimental assays demonstrated that targeting PHF6 significantly suppressed cell colony formation, migration and metastasis. Bioinformatical analysis indicated that PHF6 may regulate HIF signals and PHF6 directly interacts with HIF1α and HIF2α. PHF6 promoted HIF transcriptional activity and downstream targets levels, without altering HIF expressions. Our subsequent mechanistic investigations raised two aspects of HIF regulation by PHF6 in Bca cells. The one aspect is that PHF6 potentiated HIF binding to HRE of targets, and the another aspect is that PHF6 recruited epigenetic remodeling complex (BPTF) to sustain transcriptional efficacy. PHF6 and HIF were mutually required to activate expressions of HIF targets. Functional in vitro and in vivo assays confirmed that PHF6 depended on HIF to exert oncogenic effects under hypoxia situation. Last of all, we determined that PHF6 had well prognostic significance based on large samples, creating a biomarker for prediction and treatment of Bca.

The tumor microenvironment (TME) is recently regarged as an essential cause that influences epigenetic reprogramming [29]. The reduced O_2_ supplement represents the main characteristic of TME in Bca, and HIFs the master transcriptional regulators that control cellular responses to hypoxia condition to impact Bca progression [30, 31]. Considering that HIFs and its downstream metabolic and angiogenic remodeling contribute most to aggressiveness, metastasis, and therapy resistance of Bca, it makes sense to discover new druggable targets in HIF upstream crosstalk and downstream angiogenic and metabolic pathways [32, 33]. For instance, the researchers found that cardamonin could restrict mTOR/p70S6K axis to suppress the expressions of HIF-1α, and thereby promoted mitochondrial oxidative phosphorylation and elevated cellular reactive oxygen species (ROS) levels [34]. Besides, under hypoxia, Bca cells also depended on glutamine required for cell growth, and pharmacological inhibition of HIF-regulated SLC1A5 using V-9302 could block breast cancer cell growth, along with decreased mTOR signaling and increased ROS levels and autophagy [35]. As regard to lipid metabolism, TVB-2640, a specific FASN inhibitor targeting fatty acid (FA) synthesis, is proved to suppress proliferation and proceeded into a phase II breast cancer trial [36]. Apart from targeting downstream targets, we further speculated that whether we could abolish the HIF transcriptional activity via inhibiting the co-activators, which may suppress a wider range of downstream targets. Apparently, it is well documented that epigenetic regulators are essentially required for HIF-mediated transactivation. Previous studies have observed that CHD4, subunit of NURD complex, could increased RNA polymerase II at promoters of targets to potentiate HIF-driven transcriptional programs in breast cancer [37]. Haiquan Lu et al. found that HIF-1 recruits NANOG as a coactivator for TERT gene transcription in hypoxic breast cancer stem cells to maintain self-renewal ability [38]. Besides, the histone acetyltransferases p300, CBP, and TIP60 promote HIF-1 downstream genes via promoting acetylation of histones H3 and H4. HDACs family memebers were also uncovered to promote or inhibit HIF-1 transcriptional activity through multiple regulatory mechanisms. The role of chromatin remodelers, like BRD2/4, in HIF-1–mediated transactivation has been also demonstrated. In line with these recognitions, we also demonstrate that PHF6 functions as a co-activator of HIF and ruled out the possibilities that PHF6 could directly impact HIF levels via transcriptional regulations or post-translational modifications (PTMs) pathways. Targeting PHF6 was effective to suppress HIF downstream genes and restrict in vivo tumor growth.

Hippo signaling pathway participates in multiple biological processes of tumors. When the Hippo pathway is activated, MST1/2 phosphorylates and activates LATS1/2, which in turn phosphorylates YAP/TAZ and inhibits the activity of YAP/TAZ [39]. Inhibition of Hippo signaling is closely related to development of multiple malignancies. Most studies have shown that overexpression or activation of YAP/TAZ can promote tumor aggressiveness and is regarded as an oncogene in many solid tumors [40]. Studies have shown that YAP/TAZ regulates every step of breast cancer metastasis by directly or indirectly affecting metastasis-associated moleculars [41]. Of note, hypoxia-induced YAP activation can effectively accelerate the glycolysis process in hepatocellular carcinoma [42]. Particularly, hypoxia inhibits the Hippo signaling pathway, thereby promoting the intranuclear transfer and accumulation of YAP, and reducing the level of phosphorylated YAP in cytoplasm. Besides, activated YAP by hypoxia can directly bind to HIF1α and maintain its protein stability. Based on these knowledge, we utilized a drug screen to find that YAP inhibition could suppress PHF6 levels. ChIP-qPCR and luciferase reporter assay further demonstrated that YAP could directly bind to promoter regions of PHF6 to sustain its levels. Meanwhile, YAP depended on PHF6 to exert oncogenic impacts on Bca cells. With regard to the tight associations between PHF6 and HIF signaling, we further found that hypoxia could also depend on YAP activation to induce high PHF6 levels. Interestingly, PHF6 was not the direct downstream targets of HIF, as HIF-DKO could not alter PHF6 levels. We thus determined that PHF6 is also a hypoxia-response regulator and activated by YAP/TAZ complex.

The nucleosome remodeling factor complex (NURF) containing the SNF2L subunit catalyzes the sliding of ATP-dependent nucleosomes on the DNA template to construct the active regions of the promoter to activate target genes [43]. Previous studies have shown that the NURF complex can be recruited to the promoter region of SOX4 to participate in the maintenance of gastric cancer stem cell properties [44]. As the largest subunit of the NURF chromatin remodeling complex, the bromodomain PHD finger transcription factor (BPTF) is essential for the epigenetic regulation and gene activation via modulating DNA chromatin accessibility. BPTF maintains the self-renewal potential of glioma stem cells by promoting the transcriptional activation of the MYC gene and its downstream targets. In human melanoma models, targeting the SWI/SNF complex subunit (BRG1) and the NURF scaffolding subunit (BPTF) both inhibited tumor proliferation. Furthermore, we found that the synergistic effect of BRG1 and BPTF is required for maintaining hub gene set expressions in melanoma cells [45]. In the clear cell renal cell carcinoma model, down-regulated METTL14 induced the overexpression of BPTF. High BPTF can promote the chromatin accessibility within the promoter region of ENO2 and SRC, improving the glycolytic activity of tumor cells [46]. In the current study, we found PHF6 recruits BPTF to sustain HIF transcriptional activity. BPTF is required for PHF6-HIF binding on the HRE of targets. Based on this theory, we determined that BPTF inhibitor is effective to suppress Bca progression, but marginally works with HIF-DKO. Based on this finding, we speculated that targeting BPTF relied on HIF signals to exert antitumor effect.

We also raised several prolems in the current study that need further considerations. First of all, owing to financial limits, we failed to conduct the Chromatin Immunoprecipitation Sequencing (ChIP-Seq) and Assay for Targeting Accessible-Chromatin with high-throughout Sequencing (ATAC-Seq) to confirm the regulations between PHF6/BPTF regulators and HIF transcriptions based on the genome scale. Besides, more pre-clinical models were warranted to confirm the inhibitory efficacy of AU1 on Bca cells. Last of all, we intended to collect more samples in our hospital to validate the prognostic significance of YAP/PHF6/BPTF axis, thereby constructing HIF-related signature models for prediction.

## Conclusions

YAP activation promotes elevated PHF6 in Bca tissues, predicting poor prognosis of patients. PHF6 recruits chromatin remodeling regulator BPTF to maintain HIF transcriptional activity. Under hypoxia, PHF6 interacts with HIF1α/HIF-2α to promote their recruitment to the HRE, thereby driving breast cancer progression. Targeting PHF6/BPTF is effective in breast cancer mouse models. As a result, the YAP/PHF6/HIF axis has clinical significance and targeting this crosstalk highlights an essential strategy for Bca treatment.

## Supplementary Information

**Additional file 1: Figure S1.** Quantification of expressions of each PHF family member in control or siRNA group.

**Additional file 2: Table S1.** Summary of siRNA sequences that target each PHF family member.

## Data Availability

The data used to support the findings of this study are available from the corresponding author upon request.
