# Supplementary figures and images for: PHF6 recruits BPTF to promote HIF-dependent pathway and progression in YAP-high breast cancer
Source: J Transl Med. 2023 Mar 26;21:220. doi: 10.1186/s12967-023-04031-8 (PMC10040131; doi:10.1186/s12967-023-04031-8)

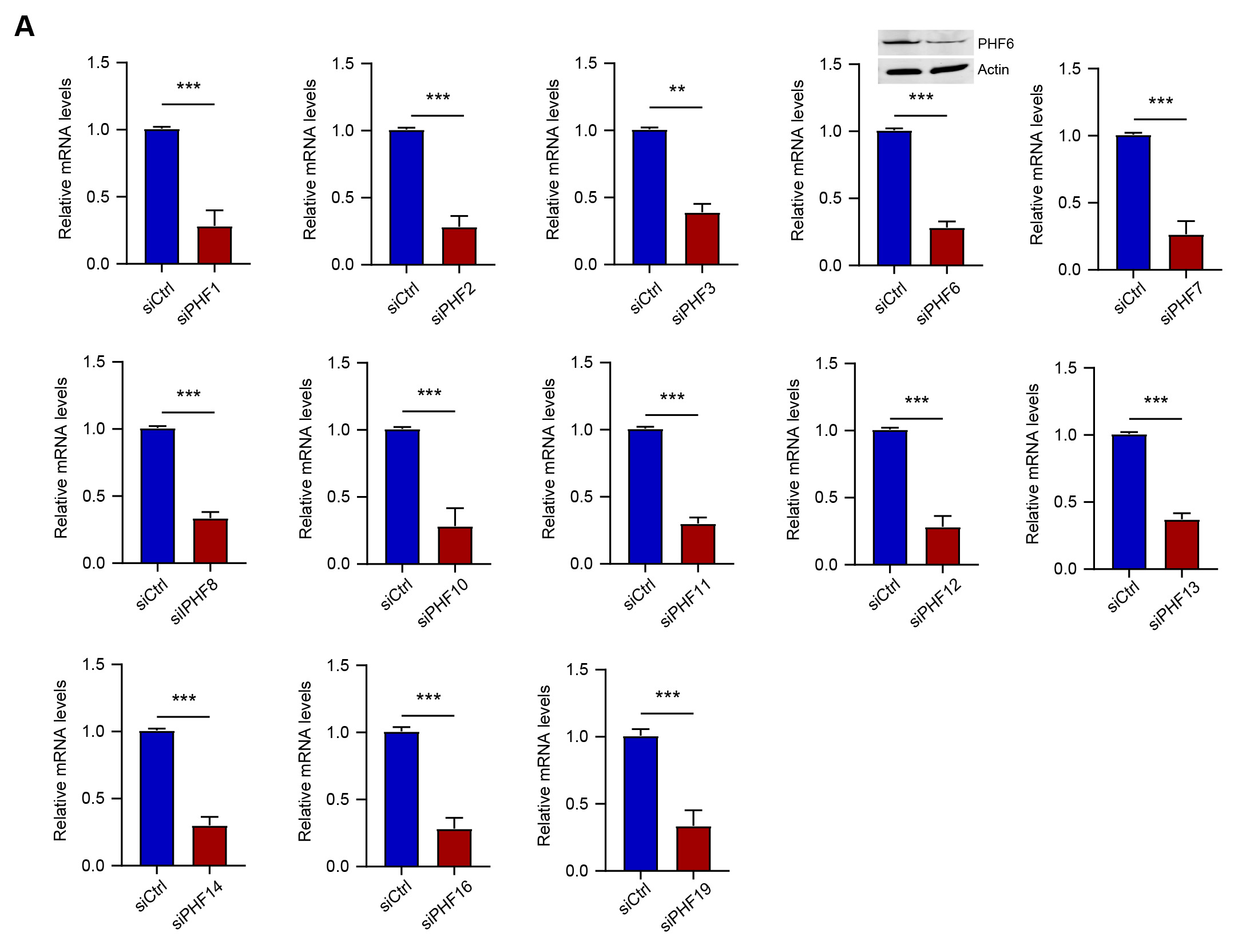

Supplement: Supplementary file 1 — Additional file 1: Figure S1. Quantification of expressions of each PHF family member in control or siRNA group. [file 12967_2023_4031_MOESM1_ESM.jpg]
