# Supplementary material for: PHF6 recruits BPTF to promote HIF-dependent pathway and progression in YAP-high breast cancer
Source: J Transl Med. 2023 Mar 26;21:220. doi: 10.1186/s12967-023-04031-8 (PMC10040131; doi:10.1186/s12967-023-04031-8)
Supplement: Supplementary file 2 — Additional file 2: Table S1. Summary of siRNA sequences that target each PHF family member. [file 12967_2023_4031_MOESM2_ESM.doc]

**Table S1 Summary of siRNA sequences that target** each PHF family member.

| **Target Gene Name** | **Sense:5’-3’** | **Anti-sense:5’-3’** |
| --- | --- | --- |
| **PHF1** | CCUCUCAUCCAGUGUGACUAUTT | AUAGUCACACUGGAUGAGAGGTT |
| **PHF2** | CCCGAGAAAUACACCUGUUAUTT | AUAACAGGUGUAUUUCUCGGGTT |
| **PHF3** | CCAGUCAAGUAGCGUUUCUUATT | UAAGAAACGCUACUUGACUGGTT |
| **PHF6** | GCACCAUAAGUGCAUGCUCUUTT | AAGAGCAUGCACUUAUGGUGCTT |
| **PHF7** | CCCACACAUCAGCAAAGCAUUTT | AAUGCUUUGCUGAUGUGUGGGTT |
| **PHF8** | CGACCCUGAUAAUAAGACCAATT | UUGGUCUUAUUAUCAGGGUCGTT |
| **PHF10** | GCCAGUAAAGUGCCUGAGUAUTT | AUACUCAGGCACUUUACUGGCTT |
| **PHF11** | CCCAAAGAUGUCGAAUAUAAUTT | AUUAUAUUCGACAUCUUUGGGTT |
| **PHF12** | CCUCUCAUCCAGUGUGACUAUTT | AUAGUCACACUGGAUGAGAGGTT |
| **PHF13** | CGGAAAUCCAAUGUUCCAGAATT | UUCUGGAACAUUGGAUUUCCGTT |
| **PHF14** | CGCAUGAUUCAAAUUCAGGAATT | UUCCUGAAUUUGAAUCAUGCGTT |
| **PHF16** | GCUCAUCUGUUCACAGUAUAATT | UUAUACUGUGAACAGAUGAGCTT |
| **PHF19** | CCUGAAAUGGACAAUCACUUUTT | AAAGUGAUUGUCCAUUUCAGGTT |
